# An exploration of the association between very early rehabilitation and outcome for the patients with acute ischaemic stroke in Japan: a nationwide retrospective cohort survey

**DOI:** 10.1186/1472-6963-10-213

**Published:** 2010-07-20

**Authors:** Hiroki Matsui, Hideki Hashimoto, Hiromasa Horiguchi, Hideo Yasunaga, Shinya Matsuda

**Affiliations:** 1 Department of Health Economics and Epidemiology Research, School of Public Health, The University of Tokyo, 7-3-1 Hongo, Bunkyo-ku, Tokyo 113-0033, Japan; 2 Department of Health Management and Policy, Graduate School of Medicine, The University of Tokyo, 7-3-1 Hongo, Bunkyo-ku, Tokyo 113-0033, Japan; 3 Department of Preventive Medicine and Community Health, University of Occupational and Environmental Health, 1-1 Iseigaoka, Yahatanishi-ku, Kitakyushu, Fukuoka 807-8555, Japan

## Abstract

**Background:**

Very early rehabilitation is expected to improve functional outcomes after stroke, although its effectiveness has not been fully evaluated. The purpose of this study was to investigate the association between very early intervention (VEI), and patient outcomes at discharge by using nationwide large data and statistical treatment for selection bias.

**Methods:**

In this study, we defined VEI as rehabilitation commencing within 3 days of stroke admission. The data were derived from a nationwide survey of acute-care hospitals conducted in 2007 for designing a reimbursement scheme and from a concurrent survey on rehabilitation services among a convenient subgroup of hospitals participating in the above survey. We included patients with a diagnosis code of ischaemic cerebrovascular disease with acute onset who underwent any rehabilitation services during hospitalisation. Surgery cases, those with no functional deficit, and those with a severe consciousness deficit upon admission were excluded. A total of 5,482 patients were enrolled from 294 hospitals. To correct for any potential selection bias, we used Friday admission as an instrumental variable (IV) and conducted a bivariate probit model analysis.

**Results:**

We found that VEI for acute stroke patients was significantly associated with a lesser degree of disability at discharge. Even after considering endogenous problems due to treatment selection, VEI improved the chance of reducing disability by 15.3% (p < 0.001). There was no significant association between VEI and in-hospital mortality, suggesting that VEI was not likely to lead to an adverse outcome.

**Conclusions:**

These data suggest that VEI may lead to a better outcome with no increase in adverse events compared to delayed rehabilitation.

## Background

Stroke remains the third largest cause of mortality in Japan, resulting in 127,000 deaths per year [1]. Although the fatality rate has decreased in the last few decades, a large number of acute survivors still suffer functional disabilities that lead to the second largest loss of disability-adjusted life years (1,078,000 person years) in Japan [2]. Ischaemic stroke patients represent approximately two-thirds of all Japanese stroke patients [3]. Recent clinical studies and meta-analyses have shown that stroke unit care is effective in reducing mortality and improving the functional outcome of stroke patients [4]. However, which specific component of stroke unit care contributes to this benefit remains unclear. Langhorne et al. showed that two-thirds of stroke units initiated rehabilitation within 3 days [5], implying the effectiveness of the very early initiation of rehabilitation (VEI) on stroke patient outcomes.

There is an ongoing large randomised controlled trial in Australia to determine the efficacy of VEI among acute stroke cases [6]. The phase 2 study of the trial (A Very Early Rehabilitation Trial for stroke (AVERT) phase 2; AVERT2) revealed the safety and feasibility of VEI [7]. The recent Cochrane systematic review of VEI, however, concluded that the efficacy of VEI remains to be established [8].

To date, many observational studies have investigated the association between early initiation of rehabilitation and patient outcomes. Hayes et al. indicated that stroke patients who received VEI were likely to have better recovery of walking ability with a shorter hospital stay, although the generalisability of this finding may be limited due to its small sample size (N = 30) that was collected in a single institute [9].

In their study of 1,716 patients admitted to several Italian rehabilitation hospitals, Musicco et al. reported that stroke patients whose rehabilitation started within 7 days of onset were likely to have better functional status 6 months after discharge [10]. This study captured a broader array of patients. Because of the nature of an observational study, however, their analysis may not fully eliminate selection bias in treatment assignment even with adjustment for patient conditions by ordinal multiple regression analysis [11].

The use of VEI treatment is dependent on the clinical characteristics of the patients and other factors, both observed and unobserved, which in turn influence subsequent health outcome, cost, and health utility. Thus, it is difficult to disentangle the effects of treatment per se from those of unobserved confounders when we use observational data. In economic studies and recently in health services research, instrumental variable (IV) methods have been used to overcome potential selection bias due to the presence of unobserved confounding factors in the observational data and to provide consistent estimates of the association between treatment and outcome for several conditions and treatments [12-15]. Simply put, IV is a randomly occurring variable associated with the administration of treatment but is not related to outcomes. By using an appropriate statistical model that includes IV, we could statistically control for selection bias and better evaluate treatment effectiveness. For example, McGuire et al. used IV methods to assess the impact of early surgical intervention on the prognosis of femoral neck fractures [14]. The indication for early surgical intervention may depend on patient characteristics, which in turn affects patient outcomes. Thus, simple comparison between only the treatment and control groups leads to biased results. McGuire and colleagues used the day of admission, which should randomly occur, as an instrument to statistically control this selection to distinguish between the two groups. Using a larger observational dataset and proper statistical methods such as IV would, therefore, allow us to determine the association of VEI treatment with patient's outcome at discharge, which shaped the purpose of this study.

## Methods

### Data source

The data used in this study were derived from a nationwide survey conducted in 2007 for developing a case-mix classification system for acute care and a related reimbursement schedule in Japan [16]. The administrative claims database that we used for this study was composed of data compulsorily submitted by the acute care hospitals under the case-mix-based reimbursement policy. Although enrolment in this newer reimbursement policy is on voluntary basis, most of the acute care hospitals in this country have joined the system. In this database, diagnoses of diseases related to major resource use during hospitalisation are recorded by physicians in charge while referring to medical charts. The reimbursement rate is set according to the registered diagnosis. The administrative claims database has several potential biases, including the possibility of over- or under-reporting due to deceived coding, which is the same as any administrative claims database. The data from cases discharged during July and December 2007 were collected in a standardised electronic format from 965 hospitals (84 academic hospitals and 891 community hospitals), which account for approximately 30% of the acute care beds in Japan. The database included patients' demographic and clinical information such as main diagnosis, co-morbidity, and provided procedures, and claim data on resource use such as the date and volume of service provision.

Another source of data was a concurrent survey on rehabilitation services among a convenient subgroup of hospitals participating in the above national survey (N = 294). The hospitals that voluntarily agreed to join the additional survey on their rehabilitation service process for patients with stroke or femoral neck fractures provided additional detailed information such as the onset date and the functional levels before onset, at admission, and on discharge.

We merged these 2 databases using the hospitals' dummy identification number, admission date, discharge date, and patient classification system code for further analysis. Patients' data were anonymously provided by the participating hospitals. These 2 projects were approved by the Ethics Committee of the University of Occupational and Environmental Health, Fukuoka, Japan.

### Data selection

#### Inclusion and Exclusion Criteria

In this study, we included those patients who were hospitalised for an acute ischaemic stroke event and had utilised rehabilitation services, including physical and occupational therapy, during their hospitalisation. To be more specific, patients who had the following international classification of diseases (ICD-10) codes (G45$, G46$, I63$, I65$, I66$, I675, I679, I693, or I978) and were hospitalised within 1 day after onset were selected from the merged database.

We excluded those who were hospitalised for more than 180 days, who had intracranial haemorrhages, and who received a surgical operation since these patients were likely to have heterogeneous conditions that may have confounded their chance of functional recovery and mortality. We also excluded those who were less likely to have a very early intervention, e.g., patients with no functional deficit upon admission (modified Rankin Scale score [mRS] = 0), patients with moderately severe disability before onset (mRS at pre-admission ≥ 4), and patients in a coma (defined using the Japan Coma Scale; JCS ≥ 100).

### Definitions of variables

We used in-hospital mortality and mRS score at discharge as outcome variables. The mRS was dichotomised as 1 when mRS was 0 or 1, and as 0 otherwise. Our dataset included the information on the day of stroke onset and the commencement of the intervention but not on details of the intervention. With the available data, we defined VEI in this study as any type of rehabilitation performed by physical or occupational therapists within 3 days after admission for acute cases admitted within 1 day after onset. Training intensity was defined as total units (1 unit = 20 minutes) of rehabilitation training during hospitalisation divided by the length of hospital stay. We also included the use of edaravone, a free radical scavenger, because it is widely used for stroke treatment in Japan, and would affect a patient's prognosis. Use of edaravone was labelled 'yes' when more than 1 dose was used during hospitalisation. Although early intervention using tissue plasminogen activator (tPA) would also influence outcome [17,18], we did not include the use of tPA since the prevalence of its use was < 1% of admitted patients. Finally, we adopted admission over the weekend as an IV in our analysis. We defined admission over the weekend as admissions made on Friday. We assumed that the chance of receiving VEI would be lower among those individuals admitted on a Friday because of the reduced manpower available for rehabilitation therapy on the following Saturday and Sunday.

We included age, gender, co-morbidity index (Charlson's Index [CI]), and pre-admission mRS as potential confounders that may affect functional and mortality outcomes. We also obtained functional levels at admission such as consciousness level (measured using the Japan Coma Scale), communication disorder, Brunnstrom stage of the upper and lower extremities, swallowing disorder, and mRS. Since these functional indices were highly inter-correlated, we used principal component analysis to reduce them into 2 independent summary scores: 'functional severity' and 'functional capability' at admission. These 2 factors had eigenvalues > 1 and represented 76.4% of the total variance. Larger functional severity scores indicated more severe functional disability, and larger functional capability scores meant better functional levels. These scores have no units.

### Statistical analysis

A widely used approach to assess the impact of VEI on outcomes would involve classifying patients according to their VEI administration and comparing outcomes between the 2 groups with risk-adjustment techniques such as multiple logistic regression models. Although commonly employed, this approach does not fully solve residual confounding and treatment selection bias due to the unmeasured characteristics of the patients and providers in the case of non-randomised observational data.

An alternative approach is the use of the IV method. IV is an observable factor that meets several conditions: 1) randomly present (exogenous); 2) associated with the likelihood of treatment selection; and 3) no direct association with the targeted outcomes [12]. The day of admission should be related to service use variation but not to outcomes except through service variation [13,14]. McGuire et al. used the day of admission as an instrument to assess the impact of early surgical intervention on the prognosis of femoral neck fractures [14]. Therefore, in our case, since rehabilitation services are less likely to be provided over the weekend [19], Friday admission is considered an effective IV.

To compare our data with those of other cross-sectional observation studies, we initially conducted an ordinal single equation probit model including VEI as a major explanatory variable with patients' characteristics at admission, training intensity, and use of edaravone as covariates for risk adjustment. We then used a bivariate probit (BVP) model to account for Friday admission as an instrument. To be specific, the first equation predicted the likelihood of VEI treatment with patient characteristics at admission as covariates and Friday admission as an instrument. The second stage equation then regressed the targeted outcomes on VEI use predicted in the first equation with patient characteristics at admission, training intensity, and edaravone use as covariates. Since the quality of rehabilitation services may vary across institutes, we also conducted a similar analysis stratified by the existence of a rehabilitation specialty as a surrogate marker of care quality.

## Results

### Patient characteristics

**Figure 1:**
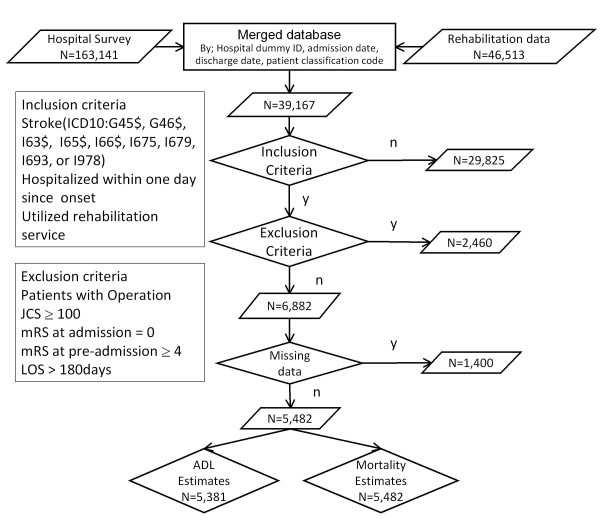
**Patient selection**. mRS: modified Rankin scale, JCS: Japan coma scale, LOS: length of stay

Figure 1 shows the patient selection process. Records of 39,167 patients were successfully merged and 6,882 patients met our inclusion criteria. Due to missing values in key variables, 1,400 observations were excluded, resulting in the availability of 5,482 patients for further analysis (Figure 1). Those patients excluded due to missing values had a 2.3% higher in-hospital mortality rate, 4.5% lower prevalence of females, and a 1 year older mean age compared with patients included in the analysis. The Friday admission rate and co-morbidity index were similar between the included and excluded subjects.

Table 1 reports patient characteristics. The average age was 73.1 (11.7) years, 39.7% were female, and 64.4% had no functional deficit at pre-admission (mRS = 0). As for clinical status at admission, 41.6% had a moderate consciousness disorder (Japan Coma Scale ≥ 3), and 17.6% showed a small functional deficit (mRS ≤ 1). VEI was administered to 74.2% of patients. Admission on Friday was observed in 829 patients (15.1%). A small functional deficit (mRS ≤ 1) that remained at the time of discharge was observed in 2,365 patients (44.0%) and the overall in-hospital mortality rate was 1.6%.

**Table 1:** Descriptive statistics of studied sample (N = 5,482)

Variable name		Descriptive analysis*		Missing from variables**
	Age (years, SD)	73.1	(11.7)	0
	Gender (Female %)	2,175	(39.7)	0
	mRS at pre-admission (0--1, %)	4,581	(83.6)	807
	mRS at admission (0-1, %)	965	(17.6)	746
	Consciousness level (JCS; 1-3, %)	3,200	(58.4)	236
	Comm. disorder, explanation (Mild, %)	4,279	(78.1)	419
	Comm. disorder, understanding (Mild, %)	4,453	(81.2)	426
	Brunnstrom stage, upper (Mild/No symptoms, %)	3,694	(67.4)	576
	Brunnstrom stage, lower (Mild/No symptoms, %)	3,896	(71.0)	579
	Swallowing disorder (Mild, %)	4,262	(77.8)	463
	Co-morbidity index (CI; 0-1, %)	2,796	(51.0)	0
	Use of edaravone (%)	3,230	(58.9)	0
	VEI (%)	4,068	(74.2)	0
	Training intensity (unit/day, SD)	1.54	(1.19)	0
	mRS at discharge (0-1, %) ***	2,365	(44.0)	818
	In-hospital mortality (%)	89	(1.6)	0
	Friday admission (%)	829	(5.1)	0

mRS: modified Rankin scale; JCS: Japan coma scale; LOS: length of stay; VEI: very early intervention; CI: Charlson's index

*: Descriptive analysis presents the number (percent) of categorical variables and the mean (standard deviation) of the continuous variables.

**: Number of missing values in patients who matched the inclusion criteria.

***: mRS at discharge was available for 5,381 patients

Table 2 reports the descriptive statistics of the studied sample divided by VEI administration. Patients who received VEI were younger and showed better pre-admission mRS, a lower functional severity score, and a worse functional capability score at admission. Patients with VEI were more likely to have a better mRS at discharge. The in-hospital mortality rate showed no significant difference in between the 2 groups. Patients who received VEI were less likely to be admitted on Friday compared to those without VEI (12.1% vs. 23.9%, p < 0.001).

**Table 2:** Comparison of those with and without VEI

	VEI (-) N = 1,414 (25.8%)	VEI (+) N = 4,068 (74.2%)	Univariate analysis (P value)*
Age in years (SD)	73.6 (11.8)	72.9 (11.7)	0.045
Gender (female)	40.2%	39.5%	0.614
mRS pre-admission			
mRS = 0	61.7%	65.3%	
mRS = 1	19.7%	19.0%	0.007
mRS = 2	8.6%	8.4%	
mRS = 3	10.0%	7.3%	
Functional severity score, mean (SD) ¶	0.24 (1.78)	0.10 (1.69)	0.005
Functional capability score, mean (SD) ¶	-0.12 (1.1)	-0.29 (0.99)	< 0.001
Co-morbidity index (CI > 1)	48.4%	49.2%	0.629
Use of edaravone	56.5%	59.8%	0.032
Training intensity (unit/day), mean (SD)	1.04 (0.84)	1.71 (1.25)	< 0.001
mRS at discharge (0-1)	40.1%	45.3%	< 0.001
In-hospital mortality	1.7%	1.6%	0.799
Friday admission	23.9%	12.1%	< 0.001

CI: Charlson's index, mRS: modified Rankin scale, VEI: very early intervention

*: Chi-square test for categorical variables and *t*-test for continuous variables.

¶: Functional severity score is the principal component of patient severity. Larger values indicate more severe patient conditions. Functional capability score is the principal component of patient functional capability. Larger values indicate better function.

### Effect of VEI on mRS at discharge

Table 3 presents the results of single probit and BVP analyses of effects on mRS at discharge. Less disability is associated with younger age, lower mRS at pre-admission, lower functional severity score, higher functional capability score, lower training intensity, and administration of VEI. VEI was significantly and positively associated with better mRS at discharge in both models. The single probit model estimation shows that VEI improved the chance of reducing disability at the time of discharge by 4.8% (p = 0.007). The BVP model showed an even larger reduction, 15.3% (p < 0.001)

**Table 3:** Results of probit model analysis to predict mRS ≦ 1 at discharge

	Single probit model		Bivariate probit model	
		
	Marginal effect (95% C.I.)		Marginal effect (95% C.I.)	
Age	-0.004**	(-0.006, -0.003)	-0.003**	(-0.004, -0.002)
Gender (female)	-0.014	(-0.046, 0.018 )	-0.010	(-0.033, 0.014)
mRS pre-admission				
mRS = 0	-	-		-
mRS = 1	-0.055**	(-0.092, -0.019)	-0.041**	(-0.068, -0.014)
mRS = 2	-0.329**	(-0.357, -0.301)	-0.220**	(-0.248, -0.192)
mRS = 3	-0.384**	(-0.406, -0.361)	-0.257**	(-0.287, -0.226)
Functional severity score¶	-0.216**	(-0.228, -0.204)	-0.155**	(-0.168, -0.142)
Functional capability score¶	0.089**	(0.073, 0.104 )	0.053**	(0.041, 0.065)
Co-morbidity index (CI > 2)	-0.017	(-0.047, 0.014)	-0.010	(-0.033, 0.013)
Use of edaravone	-0.013	(-0.044, 0.018 )	-0.009	(-0.031, 0.013)
VEI	0.048**	(0.013, 0.084 )	0.153**	(0.072, 0.234)
Training intensity	-0.015*	(-0.029, -0.002)	-0.011*	(-0.020, -0.001)
First stage regression				
Friday admission			-0.076**	(-0.092, -0.059)

N = 5,381

In the bivariate probit model, ρ = -0.30 [-0.51, 0.05], p = 0.024

CI: Charlson's index; mRS: modified Rankin scale; VEI: very early intervention

¶: Functional severity score is the principal component of patient severity. Larger values indicate more severe patient conditions. Functional capability score is the principal component of patient functional capability. Larger values indicate better function.

Training intensity had a significant negative association with mRS at discharge in both models (-0.015, p = 0.026 in the single probit model; -0.010, p = 0.031 in the BVP model).

### Effect of VEI on in-hospital mortality rate

Table 4 presents the results of single probit and BVP analyses of VEI on the in-hospital mortality rate. There was no significant association between VEI and in-hospital mortality in either model. In the BVP model, the correlation coefficient of the disturbances (ρ) was not significant for the likelihood ratio test (ρ = 0.04 [-0.59, 0.64], p = 0.91).

**Table 4:** Results of probit model analysis to predict in-hospital mortality

	Single probit model		Bivariate probit model	
		
	Marginal effect (95% C.I.)		Marginal effect (95% C.I.)	
Age (by 10 years)	0.002*	( 0.000, 0.004)	0.002	(-0.000, 0.003)
Gender (Female)	-0.000	(-0.004, 0.003)	-0.000	(-0.003, 0.002)
mRS pre-admission				
mRS = 0			-	
mRS = 1	-0.001	(-0.006, 0.003)	-0.001	(-0.005, 0.002)
mRS = 2	0.003	(-0.004, 0.010)	0.002	(-0.004, 0.008)
mRS = 3	0.003	(-0.004, 0.010)	0.002	(-0.003, 0.007)
Functional severity score¶	0.004**	(0.003, 0.006)	0.003*	(0.001, 0.006)
Functional capability score¶	0.002*	(0.000, 0.003)	0.001	(-0.000, 0.002)
Co-morbidity index (CI > 2)	0.003	(-0.001, 0.006)	0.002	(-0.001, 0.005)
Use of edaravone	0.002	(-0.002, 0.005)	0.001	(-0.002, 0.005)
VEI	0.003	(-0.000, 0.006)	0.001	(-0.013, 0.016)
Training intensity	-0.005**	(-0.007, -0.003)	-0.004*	(-0.007, 0.000)
First stage regression				
Friday admission			-0.001	(-0.002, 0.000)

N = 5,482

In the bivariate probit model: ρ = 0.04 [-0.59, 0.64], p = 0.91

CI: Charlson's index; mRS: modified Rankin scale; VEI: very early intervention

¶: Functional severity score indicates the principal component of patient severity. Larger values indicate more severe patient conditions. Functional capability score is the principal component of patient functional capability. Larger values indicate better function.

Training intensity was significantly and negatively associated with the in-hospital mortality rate in both models, suggesting that the administration of VEI reduced mortality by 0.4% in the BVP model (p = 0.031).

### Stratified analysis by availability of rehabilitation specialty services

We conducted a similar analysis stratified by the availability of rehabilitation specialty services as a proxy index of service quality. VEI was positively and significantly associated with better mRS at discharge among those with and without access to a specialty service during their hospitalisation (data not shown). Interestingly, training intensity showed a negative and significant association with better mRS at discharge among those without specialty service (marginal effect estimate = -0.281, p < 0.001), while the association was positive yet non-significant among those with specialty services (marginal effect estimate = 0.002, p = 0.797). VEI had no significant association with in-hospital mortality, while training intensity had a negative and significant relationship with in-hospital mortality in both strata (without specialty service: marginal effect estimate = -0.006, p = 0.001 in the single probit model; with specialty service: marginal effect estimate = -0.003, p = 0.014 in the single probit model).

## Discussion

Recent consensus guidelines recommend early intervention for acute stroke patients [20], but little evidence exists regarding the effectiveness of VEI for acute stroke patients. In the present study, we used IV estimation to provide insight into this important question using a large Japanese patient sample.

We found that VEI for acute stroke patients was significantly and positively associated with the chance of better mRS at discharge, even after consideration of endogenous problems due to treatment selection. Our findings were basically in agreement with those from previous observational and interventional studies.

In an observational study by Musicco et al. [10], delayed initiation of rehabilitation after 7 days since onset doubled the odds of a severe activity of daily living (ADL) deficit 6 months after admission. A simple comparison between this study and ours is not plausible because of the different case mix in terms of disease severity and different contents of rehabilitation intervention. However, this previous finding might suffer from overestimation of the causal effect of treatment since early intervention was more likely to be administered to patients with mild to moderate severity rather than to those with severe conditions such as coma.

An early report from a recently conducted randomised controlled trial (AVERT2) also showed that VEI effectively improved ADLs 3 months after onset [7]. The probability of intact ADLs 3 months after onset among treatment patients was 9.2% (-12.9, 31.2) higher than that of the control patients. Thus, the effect size was smaller than what was observed in the current study. Again, a simple comparison requires caution because of the different timing of the functional evaluation.

In the current study, a large population derived from multiple institutions was used to assess the clinical impact of VEI. We evaluated patient disability outcome at discharge since it may better reflect the effect of rehabilitation intervention during the hospitalisation period. We also adopted Friday admission as an IV to correct for treatment selection bias and unmeasured confounding effects. Friday admission was, as expected, significantly and negatively associated with the likelihood of VEI administration. In the BVP model, the correlation coefficient of the disturbances (ρ) demonstrated that the error term in the prediction model for VEI (or the first step regression) was significantly associated with that in the prediction of mRS at discharge (the second step regression), suggesting that there was an endogenous relationship between the administration of VEI and variables predicting mRS at discharge other than VEI. The positive effect of VEI on disability outcome at discharge remained significant even after consideration for endogenous problems due to treatment selection using the IV method.

On the other hand, there was no significant association between VEI and in-hospital mortality, suggesting that VEI was not likely to lead to death. A physician's decision on whether to administer VEI during the acute phase of stroke may be dependent on their assessment of the patient's need of bed rest. In this study, we excluded patients with a severe consciousness deficit. The AVERT2 study also showed that very early rehabilitation has no significant association with mortality among patients who could react to verbal commands. Thus, it seems that VEI can be safely administered to those patients with mild to moderate consciousness disturbances.

Several mechanisms are presumably involved in the positive effect of VEI on functional outcome, such as neural plasticity and cortical reorganisation enhanced by early intervention. The genes responsible for neuronal growth and synaptogenesis are expressed at their highest levels during early brain development and decline with age, but a stroke event re-initiates the increased expression of these genes for a limited period after stroke [21]. It is suggested that there is a critical period of heightened neuroplasticity and a critical time window for early rehabilitation after stroke. Furthermore, stroke model rats given early rehabilitation (5 or 14 days post-stroke) displayed significant recovery, whereas rats given delayed treatment (30 days post-stroke) exhibited little improvement [22]. Notably, early rehabilitation increased the dendritic branching of layer V cortical neurons, whereas rehabilitation that was delayed until 30 days post-stroke had no effect [22]. Early rehabilitation should also prevent disuse syndrome and related conditions such as pneumonia and decubitus ulcers, which may also lead to better chance of functional recovery.

The current findings may have important policy implications. Stroke is the second largest cause of disability adjusted life years loss in Japan. Early initiation of rehabilitation may be a promising strategy to reduce the burden of disability and subsequent long-term care costs. Besides, improved function would lead to higher quality of life and social productivity among stroke casualties. Further research on the cost effectiveness of VEI deserves academic and policy attention.

The strengths of this study include its large dataset of stroke patients from a large number of hospitals in Japan and its use of the IV method. In spite of these strengths, the current study suffers from several limitations. We used mRS as a measure of disability levels. Since mRS was originally a measure of a person's ability to self-care, it may not be a useful measure in hospitalised cases. Our dataset did not include detailed information on patients' ADLs, such as Functional Independence Measure (FIM). The mRS scores may be less sensitive to changes in functional levels during hospitalisation. In spite of this, we found a significant impact of VEI, which still supports the effectiveness of VEI to alleviate a patient's disability after acute stroke attack.

Due to a lack of information on hourly period and details of the rehabilitation therapy, we defined in this study VEI as intervention commencing within 3 days after admission, which may prohibit simple comparison of our findings with those in previous studies. Bernhardt et al. defined VEI as intervention commencing within 48 hours after stroke onset [6,7]. They also considered VEI to be any intervention delivered with the aims of reducing the time from stroke onset to first mobilisation and increasing the amount of out of bed physical activity [6,7]. Further investigation may be necessary with the standardized definition of VEI.

Due to voluntary participation, there might be a possibility of sampling bias. We compared hospitals who submitted both claim and clinical data (and were included in the analysis, N = 294) with those who submitted claim data only (N = 681). Most prominent difference was that in general, included hospitals were more likely to be high-volume large general hospitals specializing in acute care of stroke while the excluded hospitals were more likely to be smaller private facilities providing a mix of acute and chronic care services. Thus, the presented results in our analysis may be applicable only to the larger acute hospitals, and whether VEI exhibits a similar effect on patient functional outcome in a broader range of hospitals needs further investigation.

Length of hospital stay (LOS) is also an important outcome measure of treatment effectiveness. LOS is also regarded as an confounder because more severe cases may require a longer LOS. However, as OECD Health Data shows [23], Japanese hospitals exhibit significantly longer LOS values compared to hospitals in other OECD countries, which is attributed to undifferentiated hospital functions between acute and chronic care, and consequent 'hospitalisation due to social reasons' [24]. LOS is determined not only by patient medical conditions but also by patient social conditions such as availability of informal care in the household, gender roles, and regional socioeconomic resources [25]. Since information to control for patient and regional socioeconomic conditions was not present in the database, we chose not to use LOS in our analytic model.

In our analysis, every covariate was associated with mRS at discharge as expected, except for training intensity during hospitalisation, which was negatively related to mRS at discharge, a finding that requires some discussion. Kwakkel et al. reviewed 9 controlled trials and showed that intensive training was more effective for ADL recovery [26]. A Japanese epidemiological study conducted in 2003 by The Japanese Association of Rehabilitation Medicine also showed that training intensity was positively associated with improved ADLs [27]. A plausible explanation for the inconsistent findings would be the reverse causation between patient functional outcome and training intensity due to treatment selection. Ishida et al. [27] reported that patients who were expected to have better ADL recovery were less likely to receive intensive rehabilitation. Another explanation might be that training intensity was confounded by hospital characteristics. Besides, when we stratified patients by availability of specialty consulting, we observed a differential impact of training intensity on patient functional outcomes at discharge.

Furthermore, 1,400 patients were excluded from the analysis due to missing observations in key variables. The excluded patients were older, more likely to be female, and had higher in-hospital mortality rates than the included patients, which might lead to estimation bias. The majority of missing values were from patient severity variables. Three hospitals had a relatively large patient volume and a higher mortality rate, and they failed to provide these key variables. These cases would apparently form the outliers in our sample, and exclusion of these specific hospitals would not substantially affect our analytic results.

Our analysis did not examine hospital characteristics, e.g., quality of care, which would have been as influential on outcomes as patient demographic and clinical characteristics. In our analysis, we measured the quantity of rehabilitation services but did not have any information regarding the quality of services. Instead, we included specialty consulting as a surrogate marker of the quality of hospital care and confirmed a similar effect size of VEI regardless of specialty consultation. Thus, we believe that the effectiveness of VEI detected in our analysis might be robust. However, measurement of the quality of rehabilitation services deserves further analysis.

Finally, the exclusion criteria we used for the patients limit the current findings to those patients with mild to moderate severity. Whether VEI is similarly effective and safe among patients with more severe conditions remains an open question. Schweickert et al. conducted a randomised controlled trial of early intervention among sedated adults and found that patients with VEI were more likely to return to independent functional status upon hospital discharge [28].

## Conclusions

We estimated the effect of VEI among patients of acute ischaemic stroke events in Japan after correcting for treatment selection bias with instrument variable method. Our results indicated that VEI was effective for functional recovery and was safely administered to ischaemic stroke patients with mild to moderate severity.

## Competing interests

The authors declare that they have no competing interests.

## Authors' contributions

HM performed the quantitative analysis and drafted the manuscript, which was critically reviewed by all of the authors. SM and HH1 planned the study and analysed and interpreted the data. YH and HH2 analysed and interpreted the data. All authors read and approved the final manuscript.

## Pre-publication history

The pre-publication history for this paper can be accessed here:

http://www.biomedcentral.com/1472-6963/10/213/prepub
